# Voyages in Vacation.—XVII

**Published:** 1889-02-02

**Authors:** 


### Voyages in Vacation.—XVII.

### By One in Search of Restful Restoratives.

*(Continued from page 271.)*

### SWEDEN AND STOCKHOLM.

Stockholm, standing as it does mainly upon three islands, with portions of the town lying in the plain or situated upon rocky hills, with abundance of rich foliage of all kinds, and water and islands in all directions as far as the eye can penetrate, has much to justify the description once given of it as the Venice of the North. Others who have penetrated into all parts of the town and neighbourhood have also come to compare it to Geneva, and this latter description is true of portions of the city and its bridges, the resemblance being in some respects remarkable. We have said that parts of the town are situated in the plain and parts on the rocky hills which surround it, the approach from the former to the latter being by steam hoists or lifts of considerable altitude, from the summit of which excellent views may be enjoyed. The air of Stockholm is exhilarating, and in the summer and early autumn there are few places in the world which offer greater attractions to the visitor than the City of the Islands, the queen of excursion centres, and the home of everything which conduces to health, to sport, and to good company. In the winter there is much to be done, and outdoor and indoor amusements may be had in great variety and some novelty. It must further be remembered that Stockholm is relatively inexpensive, and that one may reside here for some months without excessive expenditure, although enjoying the best of society and amusements which the place has to offer. Of society, it must be said that the Swedes are most sociable and hospitable people, frank, companionable, and generous. Of amusements, it may be explained that there are various entertainments, including two circuses, in which the ring is converted into a stage; and during a change of scene a curtain is drawn up from the bottom, which encloses the ring, and enables the necessary re-arrangements to be carried on out of sight of the audience. This kind of entertainment, with its method of changing the scenes, was wholly novel as applied to a circus, so far as our experience went. The opera is maintained in efficiency; the performances are above the average, and the artistes engaged often of the very first rank, It was near the opera house—that is, from the balcony of the Grand Hotel—that Christine Nilsson once sang for the amusement of her fellow-townsmen and townswomen, when the crush was so great that many people lost their lives.

The drives and walks in the neighbourhood of Stockholm offer many attractions; the scene is ever changing, and the novelty of much that is encountered enhances the pleasure and lessens the fatigue of such rambles. Water-bound as Stockholm is, it can well be imagined that water excursions are the greatest of its attractions, and we think the best. During the summer a selection from some fifty different routes may be made every day, and not the least interesting portion is the start, because it enables one to realise what pride the captains take in these passenger steamers, the wonderful pace at which the boats travel, and the perfect control under which the captain has not only the machinery but every portion of the vessel. Englishmen will be familiar with the vacuum brake, and the pace at which it enables the engine-driver to bring a train into a station, because he can stop it with certainty almost within it own length. So with the Stockholm steamers, which are small enough to permit the captain to take them within a few yards of the shore at full speed, then to reverse, and so to bring them up to the anchorage more quietly than it is possible to conceive. Certainly all visitors should notice how the steamers are manipulated, for it is one of the most interesting and remarkable of sights. It is the custom for the inhabitants to dine in summer and autumn at restaurants situated on the rivers, and often commanding excellent views. The chief of these is the Hasselbacken, where there is music in the afternoon, and a sort of Alhambra entertainment in the evening; the visitors dining in the garden when the weather permits. This restaurant is famous for its wines and cuisine, and our experience of it left little, if anything, to be desired. There is also a deer park within two miles of the city. It contains many attractive walks, where true lovers may be seen in all directions and possibly at all times. Many of the townspeople have private residences in this park, which is delightfully situated, and is altogether a charming place, to be happily remembered by those who have seen it.

Nothing could exceed the simplicity, not to say the plainness of the surroundings of the King and the Royal Family of Sweden. The palaces present a marked contrast to those of Russia, and for this reason, that although the apartments are sufficiently spacious for the purposes to which they are devoted, they are not of very striking appearance, and are of little interest to a stranger. In the ball-room a new feature has been introduced to give the appearance of height and spaciousness, which must be declared a complete success. The ceiling and cornices have been painted by an artist or artists of celebrity to represent spacious corridors leading into magnificent apartments. So well has this idea been carried out, assisted, as it is, by mirrors placed round the room, that anyone seated on one of the side ottomans gets the impression that the extent of the saloon is unlimited, and the guests well-nigh innumerable. This curious effect is, we believe, unique, as it is held to be by those in charge of the palace. The chief impression gained in visiting the palace at Stockholm, or the summer residence of the King at Karlberg, is the freedom of intercourse which custom sanctions and encourages between prince and people. Thus a visitor may wander through the grounds and stables, and even into the Palace of Karlberg itself, almost unchallenged; whilst the extensive park is quite open, and may be traversed by anyone throughout its whole length. Indeed, some of our party when at Karlberg met a very pleasant gentleman, in ordinary costume, who invited them into the palace and showed them many objects of interest. It turned out afterwards that this kindly host was none other than the King himself. This incident graphically illustrates the frank cordiality to Englishmen which one experiences throughout the whole of Sweden and Norway, as well as in Denmark. The existence of this feeling may be taken as some evidence of the kinship which exists between nations, and as one more proof that blood is thicker than water.

Another feature of the country which is worthy of study by those interested in the "employment of women" question is the variety of work entrusted to women in Sweden. On entering a bank, for instance, one notices that a fair proportion of the clerks are women, and on inquiry we found that this custom has prevailed for many years, and has been found to work exceedingly well. They make excellent bookkeepers, are reliable, faithful and honest, whilst their handwriting and figures leave nothing to be desired. Similarly, all the attendants on board the canal boats are women, the reason being, as we understood, that there is a much larger proportion of women than men in the country, and that they are thus trained to pursue many callings which are still held at home to be the sole prerogative of the sterner sex. We believe that many who advocate the cause of women in this country would find much to interest, instruct, and help them if they were to visit Stockholm and study the many phases of this question to be found there. Of course it is not to be supposed—if indeed it is not matter for thankfulness—that the exhibitions, museums, &c., of Sweden and Norway are either so large or so important as those nearer home. Stockholm is, however, a good place to buy furs of first quality at a moderate price. It may interest ladies to hear that, although seal skins have to be sent to England to be dyed and otherwise prepared for use, they can be bought in Sweden at something like 75 per cent. less than the prices charged for identical furs in London to-day. Stockholm is also famous for old armour and weapons of various kinds, imitations of which are manufactured here in large quantities, the faithful reproduction of ancient designs; he skill manifested in the execution of the work, and the surprising cheapness of much of it, make the manufactory in Stockholm very interesting and highly successful, and its productions are sent to all parts of the world.

Stockholm has good reason to be proud of the extent and variety of its charitable institutions. Not only does it possess hospitals and asylums, but it has recently erected a foundling institution of an extent and importance which place it in the first rank, and make it probably one of the most complete and well-arranged charities of its class. The asylums are not nearly so well found or administered as the hospitals, and it appears that the managers do not yet appreciate the importance of providing adequate amusements and occupation for the patients, nor do they place sufficient value upon the tasteful decoration and arrangement of the day rooms and dormitories. Nursing in Sweden has not yet been raised to the dignity and importance of a profession. The women employed are chiefly drawn from the humbler ranks of society. Their wages are small, and the relative number of women employed to patients is far below what our experience shows to be necessary to the proper nursing of the classes of disease which have to be treated in hospitals. The nurses did not appear to have had any proper training, and although individually intelligent and sympathetic, we believe they at present labour under serious disadvantages as compared with their sisters in England. It is a curious sight to English eyes to meet the surgeons and dressers going round the wards. These gentlemen wear white holland blouses long enough to extend to the feet, which have side pockets, and are fastened with buttons down the front, and a band round the centre. Their presence in this costume reminded one more of a large meat-stall than of anything else, and we felt certain that the patients of an English hospital would neither like nor submit to the wearing of such an uniform by the medical officers in whose care they might be placed.

*(To be continued.)*

